# Draft Whole-Genome Sequence of the Type Strain *Bacillus horikoshii* DSM 8719

**DOI:** 10.1128/genomeA.00641-16

**Published:** 2016-07-14

**Authors:** Ismael L. Hernández-González, Gabriela Olmedo-Álvarez

**Affiliations:** Departamento de Ingeniería Genética, Centro de Investigaciones y Estudios Avanzados del Instituto Politécnico Nacional, Unidad Irapuato, Irapuato, Mexico

## Abstract

Members of the *Bacillus* genus have been extensively studied because of their ability to produce enzymes with high biotechnological value. Here, we report the draft of the whole-genome sequence of the type strain *Bacillus horikoshii* DSM 8719, an alkali-tolerant strain.

## GENOME ANNOUNCEMENT

The study of *Bacillus* spp. isolated from alkaline environments has taken a high importance due to their application in biotechnological processes. Species such as *Bacillus horikoshii* have been studied due to their ability to produce enzymes of biotechnological interest. The type strain *B. horikoshii* DSM 8719 has been reported as isolated from soil (1), and many bacteria identified as strains of *B. horikoshii* (based on the 16S rRNA sequence) have been isolated from marine environments and sometimes have been found associated with marine organisms (2). In some cases, these strains have shown the capacity to produce metabolites with antimicrobial activities. Others have the ability to produce alkaline enzymes important to industry (3, 4). The sequenced genome of the type strain *B. horikoshii* DSM 8719 will provide important data not only for biotechnology but also for understanding the evolution of the *B. horikoshii* group*.*

The whole-genome sequencing of type strain *B. horikoshii* DSM 8719 was done through a hybrid strategy. The type strain was obtained from ATCC. The genome was sequenced using a 454 GS-FLX platform and Illumina mate-pair library. The raw 454 reads were processed using Newbler software, while the Illumina reads were processed by quality and length employing the FASTX toolkit package (http://hannonlab.cshl.edu/fastx_toolkit/index.html). *De novo* assembly was carried out using MIRA3 (5) and Velvet (6) software. The assembly produced 67 contigs (>1,000 bp) that comprise 4,677,891 bases with a G+C content of 41.3%. The genome annotation using RAST (7) identified 4,892 open reading frames (ORFs), 84 tRNAs, and 22 rRNAs. Nearly 27% of the 4,892 ORFs correspond to hypothetical proteins (1,336 ORFs).

Based on the 16S rRNA sequence, the closest strain to *B. horikoshii* is *Bacillus* sp. m3-13 (ACPC01000000) (8). From the genome sequence annotated with RAST, we obtained ORFs that were screened for secondary metabolites employing antiSMASH version 3.0.4 (9). The antiSMASH software identified 7 possible gene clusters corresponding to terpene gene synthase (2 clusters), thiopeptide-lantipeptide (2 clusters), thiopeptide (1 cluster), T3pks (1 cluster), and siderophore (1 cluster). Both thiopeptides-lantipeptide clusters correspond to lantipeptides of class I. Additionally, enzymes of potential interest were readily identified, such as an alpha-amylase, one of which had been reported in another *B. horikoshii* strain (accession no. WP_063562491, with 93% identity) and a subtilin-related peptidase S8 (WP_063562468, with 92% identity). A large number of known and hypothetical proteases were recovered with the MEROPS database (10). In addition to its importance as a producer of compounds with biotechnological value, *B. horikoshii* and its relatives represent one new group inside the *Bacillus* genus*.* The knowledge obtained from the study of the genome of this organism will shed light on the evolution of the *Bacillus* genus*.*

### Nucleotide sequence accession numbers.

This whole-genome shotgun project has been deposited in DDBJ/EMBL/GenBank under the accession number LQXN00000000. The version described in this paper is the first version, LQXN01000000.
